# Molecular Affinity of Mabolo Extracts to an Octopamine Receptor of a Fruit Fly

**DOI:** 10.3390/molecules22101677

**Published:** 2017-10-24

**Authors:** Francoise Neil D. Dacanay, Ma. Carmina Joyce A. Ladra, Hiyas A. Junio, Ricky B. Nellas

**Affiliations:** Institute of Chemistry, University of the Philippines Diliman, Quezon City 1101, Philippines; fnd06101995@gmail.com (F.N.D.D.); carminaladra@gmail.com (M.C.J.A.L.); hiyas.junio@gmail.com (H.A.J.)

**Keywords:** *Diospyros discolor* (Willd.), *Drosophila melanogaster*, ensemble docking, untargeted metabolomics, flexible receptor-ligand docking

## Abstract

Essential oils extracted from plants are composed of volatile organic compounds that can affect insect behavior. Identifying the active components of the essential oils to their biochemical target is necessary to design novel biopesticides. In this study, essential oils extracted from *Diospyros discolor* (Willd.) were analyzed using gas chromatography mass spectroscopy (GC-MS) to create an untargeted metabolite profile. Subsequently, a conformational ensemble of the *Drosophila melanogaster* octopamine receptor in mushroom bodies (OAMB) was created from a molecular dynamics simulation to resemble a flexible receptor for docking studies. GC-MS analysis revealed the presence of several metabolites, i.e. mostly aromatic esters. Interestingly, these aromatic esters were found to exhibit relatively higher binding affinities to OAMB than the receptor’s natural agonist, octopamine. The molecular origin of this observed enhanced affinity is the π-stacking interaction between the aromatic moieties of the residues and ligands. This strategy, computational inspection in tandem with untargeted metabolomics, may provide insights in screening the essential oils as potential OAMB inhibitors.

## 1. Introduction

In the search for novel pest control compounds, plant essential oils become an interesting topic as natural products-based pesticides, also known as “biopesticides”, are safer than synthetic compounds available in the market. Essential oils are mixtures of metabolites that are believed to have a repugnant effect on insects [1,2,3,4,5]. Mabolo, as it is more popularly known, merits its inclusion in the genus *Diospyros* (“divine fruit”) because of the edible reddish-orange fruit with velvet fur and a characteristic smell attributed to the presence of several volatile metabolites. Diverse compounds that were characterized to be responsible for the distinct aroma of the fruit were identified to be *n*-alkyl esters of *n*-butyric and benzoic acids as well as methyl and benzyl esters of salicylic acids [6]. *Diospyros discolor* (Willd.) (syn. *Diospyros blancoi* (A. DC.), *Cavanillea philippensis* (Desr.)) [7], was found to contain flavonoids, tannins, alkaloids, gum, and reducing sugar [8]. Other parts of the plant were reported to have been used in traditional medicine in some countries. Bengali folkloric use of the juice from bark and leaves included use as an antidote for spider and snake bites, an eyewash and cleanser for external ailments such as eczema, relief for gastrointerstinal complaints, a soothing remedy for cardiovascular problems, and a cure for diabetes [9]. In the Guianas, young leaf decoction is utilized as a remedy for hypertension, heart ailments, and diabetes [10].

In the search for novel pest control compounds, plant essential oils become an interesting topic as natural products-based pesticides, also known as “biopesticides”, are safer than synthetic compounds available in the market. Essential oils are mixtures of metabolites that are believed to have a repugnant effect on insects [1,2,3,4,5]. Mabolo, as it is more popularly known, merits its inclusion in the genus *Diospyros* (divine fruit) because of the edible reddish-orange fruit with velvet fur and a characteristic smell attributed to the presence of several volatile metabolites. Diverse compounds that were characterized to be responsible for the distinct aroma of the fruit were identified to be *n*-alkyl esters of *n*-butyric and benzoic acids as well as methyl and benzyl esters of salicylic acids [6]. *Diospyros discolor* (Willd.) (syn. *Diospyros blancoi* (A. DC.), *Cavanillea philippensis* (Desr.)) [7], was found to contain flavonoids, tannins, alkaloids, gum, and reducing sugar [8]. Other parts of the plant were reported to have been used in traditional medicine in some countries. Bengali folkloric use of the juice from bark and leaves included use as an antidote for spider and snake bites, an eyewash and cleanser for external ailments such as eczema, relief for gastrointerstinal complaints, a soothing remedy for cardiovascular problems, and a cure for diabetes [9]. In the Guianas, young leaf decoction is utilized as a remedy for hypertension, heart ailments, and diabetes [10].

The presence of a strong, characteristic pungent odor, which appears to repel ants, makes mabolo a potential source of small molecules that could be used for pest control. Previous studies have shown that profiled volatile compounds were able to act as a plant’s defense mechanism against herbivory [11,12,13,14,15]. This specific insect-plant interaction was observed to be mediated by the release of plant volatiles acting as allomones, kairomones, or synomones [11,12,13,14,15]. However, the effective use of plant volatiles as biocontrol agents requires a molecular level understanding of the effect of secondary metabolites to insect behavior. Identification of the active volatile constituents and their mode of action with respect to their biochemical targets are necessary to predict their activity and probable cross-reactivity in insects. A strategy to address this challenge is through metabolomics and in silico techniques.

Octopamine receptors (OAR) are signaling proteins that belong to the rhodopsin-like family of G-protein coupled receptors (GPCR) [16]. A conserved structure is observed among the rhodopsin-like GPCRs, mainly the transmembrane domain consisting of seven α-helices [17]. OARs are involved in a variety of important physiological functions such as mediation of intracellular calcium levels and modulating the level of adenylyl cyclase activity [18,19]. A specific OAR in the *Drosophila melanogaster* species is found to be important in female ovulation among other functions [20]. This protein known as the octopamine receptor in mushroom bodies (OAMB) is preferentially expressed in mushroom bodies or the part of the insect’s brain that is involved in olfactory learning and memory of insects [21]. OAMB was also found to affect production and the release of cyclic adenosine monophosphate (cAMP) and Ca${}^{2+}$, respectively [22]. These biologically important functions make the octopamine receptor system a possible alternative insecticide target, as most commercial pesticides target the γ-amino butyric acid (GABA) and the acetylcholinesterase systems [23,24]. As such, finding an inhibitor for this particular protein may potentially lead to effective pest control agents.

In this work, essential oils extracted from a mabolo fruit were characterized using gas chromatography mass spectrometry (GC-MS) to create an untargeted metabolite profile, i.e., we did not focus on a particular feature, such as mass–charge ratio, to analyze. Moreover, since there are no available crystal structure of octopamine receptors, we resorted to template-based homology modeling to obtain a rational three-dimensional model of the receptor. Molecular dynamics (MD) simulations were then performed to acquire an ensemble of conformations to be used in molecular docking studies. Instead of performing a series of MD simulations on all receptor-ligand systems, which is computationally demanding, we utilize a method wherein we perform MD on the apo (non-liganded) protein and obtain snapshots of different conformations to be screened using docking studies. Faster than running multiple MD simulations, this protocol aims to circumvent the problem of rigid-receptor molecular docking by obtaining binding poses of ligands to different conformations of the same protein. Although this procedure is unable to determine ligand effects upon binding, it is quite useful in sampling different docking poses and identifying possible binding sites on different protein conformations. Ultimately, this study aims to provide useful insights on the OAMB-metabolite interactions in the search for potential biopesticides using untargeted metabolite profiling in tandem with in silico techniques.

## 2. Results and Discussion

### 2.1. Essential Oil Extracts

**Figure 1:**
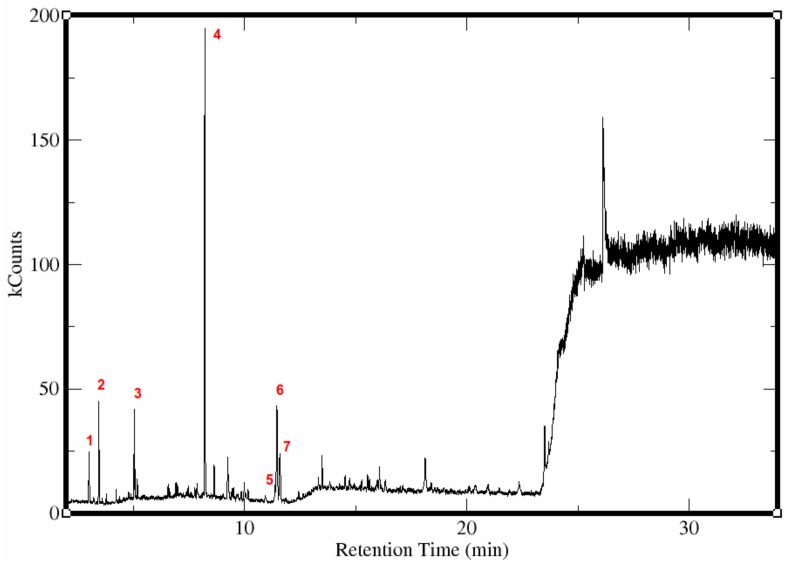
Chromatogram of essential oils extracted from mabolo fruit using Gas Chromatography-Mass Spectrometry. The peaks are labeled according to each compound identified. (1) Methyl butanoate, (2) Ethyl butanoate, (3) Butyl butanoate, (4) Methyl benzoate, (5) Butyl benzoate, (6) Benzyl butanoate, (7) Benzyl alcohol.

Untargeted metabolite profiling of the *n*-hexane extract of the mabolo essential oil yielded several peaks as shown in the chromatogram (Figure 1 and Table 1). Metabolites identified are esters, specifically butanoates (methyl, 5.6 %; ethyl, 8.5 %; butyl, 6.0 % & benzyl, 15.2 %), and benzoates (methyl, 53.4 % & butyl, 1.4 %) and benzyl alcohol (9.8 %). Metabolites detected from the sample is comparable with previous results [25,26,27,28,29]. Butanoates were detected at low abundance, possibly due to hydrolysis reaction as suggested from previous studies [26,30]. Alkyl butanoates are hydrolyzed to produce free butyric acid, which gives the olfactory signature of mabolo during steam distillation [26]. Only the corresponding benzyl alcohol was detected among the possible alcohol hydrolysis products. Furthermore, the butanoates and benzyl alcohol were previously detected from yellow passion fruit (*Passiflora edulis* (Sims. f.). *Flavicarpa* Degener) and were identified to be odor active constitutents [31]. Butanoates are decribed to have fruity, sweet and floral aroma, while benzyl alcohol has herbal, moldy and roasted seed aroma [31].

Methyl benzoate is the most abundant ester present in the sample. Compared with aliphatic esters, the hydrolysis of organic esters, such as benzoates, occurs much slower, which could account for the higher percentage of benzoates versus butanoates [30]. Methyl benzoate is one of the most abundant phenylpropanoid-derived volatile emitted from different plant parts and sources [32,33,34]. It is documented to have attractant activity to *Hylastinus obscurus* (clover root borer) [33] but potential repellent activity to *Apis mellifera* (honeybees) [35] and strong repellency to several pestiferous social wasps (yellow jackets, *Vespula pennyslvanica* and paper wasps, *Polistes dominulus*) [36].

Previously, methyl butanoate and methyl propionate were reported to be more effective in eliciting a response in sensilla trichodea of mosquitoes than the ethyl esters of these compounds at similar stimulus intensities [37]. These results showed that methyl esters attracted more gravid female mosquitoes than did the ethyl esters of the same compounds—propionic and butyric acids—when used in 0.1% aqueous solution. Moreover, sensilla trichodea’s response is relatively specific for chemical substances reported to be oviposition attractants by mosquitoes [37]. In addition, yellow jackets were found to be attracted to the combination of butyl butanoate with acetic acid as well as to isobutanol and heptyl butanoate with acetic acid [38].

### 2.2. Protein Model Verification

Due to the absence of an OAMB crystal structure, homology modeling was performed. Here, we used the web-based server, GPCR-I-TASSER, to construct a full-length protein model of OAMB. Top templates used for modeling OAMB includes the human M2 muscarinic acetylcholine receptor (PDB: 3UON, 467 residues) [39], human $\beta_{2}$-adrenergic receptor (PDB: 3D4S, 490 residues) [40], human cannabinoid receptor CB1 (PDB: 5TGZ, 452 residues) [41], rat neurotensin receptor NTS1 (PDB: 4GRV, 510 residues) [42], human A${}_{2A}$ adenosine receptor (PDB: 3EML, 488 residues) [43], and a human membrane protein/hydrolase (PDB: 5D6L, 500 residues) [44].

The aforementioned proteins were used as templates to create five OAMB models. Among the five resulting structures, the best model was selected using C-score as the standard. This metric measures the quality of the models based on the template alignments, and it typically has values ranging from −5 to +2. The best OAMB model had a C-score value of −2.74. The reason for this low score could be attributed to the low sequence identity and a large difference in length between OAMB (645 residues) and the templates (452 to 510 residues).

**Figure 2:**
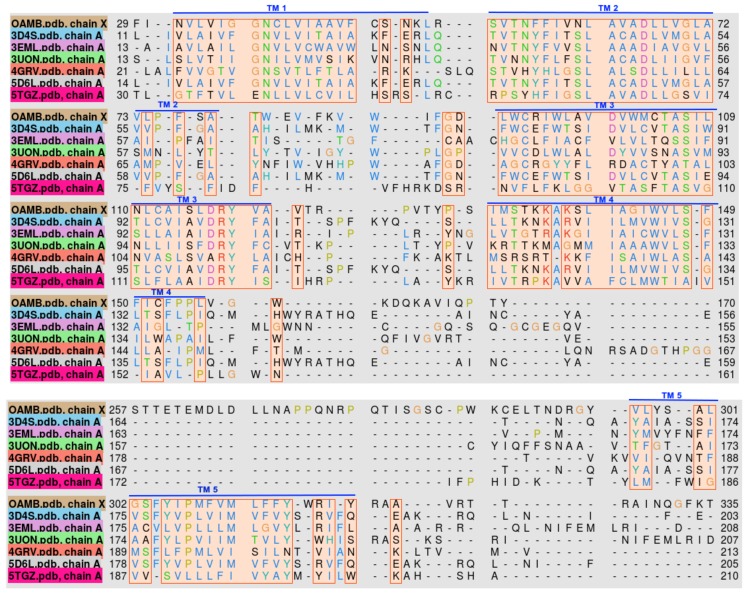

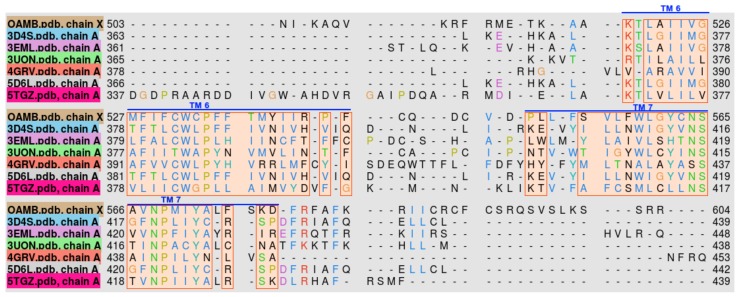
Transmembrane helix sequence alignment of *Drosophila melanogaster* OAMB and the top templates used in the homology modeling. Helix numbers are specified by the overbars.

The homologs used for modeling were between 18% and 22% identical with OAMB. After performing multiple sequence alignment of OAMB and the templates, it was found that most of the conserved sequences are located in the transmembrane region of the proteins, as shown in Figure 2.

Comparison of the protein structures were done by performing 3D alignment of OAMB and the homologs. Heavily conserved structural domains are found in the transmembrane region, which is an evolutionary characteristic of G-protein coupled receptors. Large structural discrepancies can be observed in the loop regions, i.e., intracellular and extracellular loops. This major problem in protein structure prediction can be attributed to sequence unalignment of highly varying sequences within a given structural motif.

### 2.3. Ensemble Docking

A structural ensemble was obtained from the molecular dynamics simulations of the non-liganded OAMB. This was used to study the ensemble binding affinity and binding sites of various ligands at different conformations of the protein. After obtaining ligand structures and an ensemble of protein conformations, molecular docking was performed using AutoDock Vina. The putative ligand, octopamine, along with the isolated compounds (Table 1), i.e., benzyl alcohol, butyl benzoate, benzyl butanoate, butyl butanoate, ethyl butanoate, methyl benzoate, and methyl butanoate, were docked to obtain the binding free energy of the complexes formed between the receptor and the ligands of interest. The inhibition constant $K_{i}$ could be derived from the binding affinities using the formula $K_{i}=exp\left(\frac{\Delta G}{RT}\right)$. Since there is an inverse relationship between binding affinity and $K_{i}$, it follows that the compound with the best binding affinity will have the lowest concentration requirement to inhibit OAMB.

Ensemble docking results (Table 2) show that the compound with the highest mean binding affinity is benzyl butanoate with a value of −6.03 ± 0.09 kcal·mol${}^{-1}$, followed by butyl benzoate, which has a comparable affinity of −6.02 ± 0.01 kcal·mol${}^{-1}$. Octopamine, the putative ligand, possess an average binding affinity of −5.18 ± 0.07 kcal·mol${}^{-1}$. Moreover, methyl benzoate, the most abundant compound isolated, had a binding affinity of −5.61 ± 0.07 kcal·mol${}^{-1}$. Benzyl butanoate, butyl benzoate, and methyl benzoate are slightly better than octopamine. Other extracted compounds are inferior than octopamine, i.e., benzyl alcohol, butyl butanoate, ethyl butanoate, and methyl butanoate, with binding affinities of −4.93 ± 0.06, −4.88 ± 0.06, −4.41 ± 0.06, −4.06 ± 0.05, in kcal·mol${}^{-1}$ respectively.

**Figure 3:**
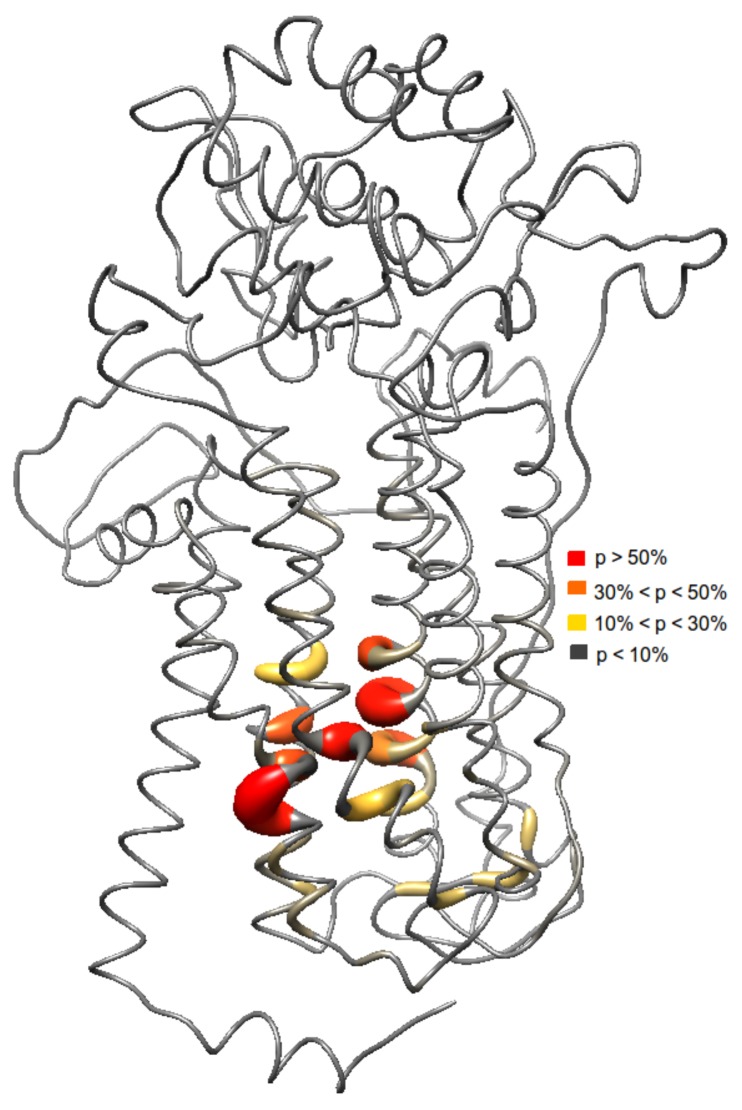
Different binding sites were observed from the ensemble docking since the ensemble is a collection of different conformations of the protein. Here, probability (p) of the ligands to interact with several residues is shown.

Results from the docking studies suggest that an aromatic motif induces favorable binding. Investigating the binding sites of the ligands reveal a similar set of amino acids (Figure 3). The probability of each ligand to bind in a specific site was also analyzed. Here, the most frequent binding region includes the residues Val69, Val99, Met103, Cys104, Ser107, Trp532, Gly559, Trp560, and Asn562. Based on the ensemble docking, these residues have >50% probability of ligand interactions. Furthermore, these residues belong to the transmembrane helices 2, 3, 6, and 7. Other residues that interact with the ligands were Ala65, Asp66, Trp80, Trp96, Leu97, Asp100, Val101, Trp159, Trp285, Cys287, Glu288, Phe304, Phe535, Phe536, Arg542, and Phe556 of the extracellular loops 2, 3, and 4 and transmembrane helices 2, 3, 4, 5, 6, and 7 with ligand interaction probabilities of 10–50%. Aromatic residues in the binding regions, i.e., Trp80, Trp96, Trp159, Trp285, Phe304, Trp532, Phe535, Trp560, Phe536, and Phe556 interacting with the phenyl ring of the aromatic ligands (π−π stacking interactions) seems to be the origin for the observed binding affinity.

Further analysis shows that there are two possible binding pockets. The site with the higher probability of ligand binding (>50%) consists of Ala65, Ala66, Val69, Trp96, Leu97, Val99, Asp100, Val101, Met103, Cys 104, Ser107, Trp532, Phe535, Phe536, Gly559, Phe556, Tyr560, and Asn562. The second binding pocket with a lower ligand interaction probability (<50%) consists of Trp80, Trp159, Trp285, Cys287, Glu288, Phe304, and Arg542 of the extracellular loops 2, 3, 4, and transmembrane helices 4 and 5. The aforementioned residues in the first binding pocket is located at the core of the transmembrane domain in helices 2, 3, 6, and 7.

The fruit of mabolo is a good source of aromatic compounds (Table 1). The most abundant compound methyl benzoate, with a 53.4% percent abundance in the fruit, has a better binding affinity than octopamine. Interestingly, benzyl butanoate, the best binding ligand is also the second most abundant compound in the fruit with a percent abundance of 15.23%. This indicates that the mabolo fruit is an excellent source of pest control compounds.

There are very few available studies regarding OAMB. Various works show that OAMB is known to be crucial for olfactory learning and motor control [21,45]. Moreover, OAMB affects female fruit fly ovulation [20], which could possibly be impaired by antagonistic activity against OAMB. Hindering the function of this receptor could potentially cause a decline in fruit fly reproduction [20] and erratic behavior, leading to a decrease in crop produce damage. The antagonistic effect of the mabolo fruit extracts will need to be tested via live insect assay to empirically determine the effects of the compounds, i.e., attractants, repellents, and/or sterilizer (inhibit egg fertilization) and obtain a feedback for the modeling experiments. Thus, this study may well serve as a guide for the synthesis of potent biopesticides by rational design of functional derivatives that possess better binding affinities than the isolated compounds.

## 3. Materials and Methods

### 3.1. Extraction of Essential Oil Components

Fruit samples were collected from Barangay Patalan, at the town of Paniqui in the province of Tarlac, Philippines. Fruits were homogenized with a blending machine (Cherenz Global Manufacturing Inc., Metro Manila, Philippines) prior to steam distillation. Collected distillate was extracted with *n*-hexane at a 1:1 ($\frac{v}{v}$) ratio. The organic extract was concentrated in vacuo with a rotary evaporator (Keison International Ltd., England, United Kingdom). Constituents of the organic extract were profiled by way of gas chromatography mass spectrometry with Varian GC-MS (GC: Varian Inc., Middelburg, The Netherlands; MS: Varian Inc., Walnut Creek, CA, USA). Zebron ZB-WAXplus (30 m length, 0.25 mm inside diameter, 0.25 μm film thickn ess) column was used with helium (99.999%) as carrier gas at a flow rate of 1.0 mL·min${}^{-1}$. Injector temperature was set at 240 ${}^{\circ }$C. Initial column temperature was at 50 ${}^{\circ }$C for 2 min, increased at 20 ${}^{\circ }$C·min${}^{-1}$ to 150 ${}^{\circ }$C, held for 5 min, increased at 40 ${}^{\circ }$C·min${}^{-1}$ to 200 ${}^{\circ }$C, held for 10 min, then finally increased at 60 ${}^{\circ }$C·min${}^{-1}$ to 250 ${}^{\circ }$C, held for 10 min. Total analysis time for each run is 45.0 min at column flow rate of 1.0 mL·min${}^{-1}$. MS parameters were set as follows: scan range from 50 to 500 m·e${}^{-1}$ with electron ionization at 70 eV and a scan rate of 0.41 sec per scan.

Data acquisition and analysis were done with Varian MS Workstation Software version 6.9.1 (Varian Inc., Walnut Creek, CA, USA) with MS Data Review, Automated Mass Spectral Deconvolution and Identification System II (AMDIS II), and National Institute of Standards Mass Spectral Search Program (NIST MS) softwares. Identification of secondary metabolites was made using NIST Mass Spectral Search Program Version 2.0f (National Institute Standard and Technology, Scientific Instrument Services Inc., Ringoes, NJ, USA).

### 3.2. Receptor Modeling and Ligand Preparation

The complete amino acid sequence of OAMB was obtained from the UniProt database (Uniprot ID: Q7JQF1) [46]. Due to alternative splicing, two isoforms of the protein were accessible. In this study, we used the isoform (Uniprot ID: Q7JQF1-1) identified as the canonical sequence (645 amino acid residues) over the other isoform (Uniprot ID: Q7JQF1-2, 637 amino acid residues) whose sequence differs from the canonical isoform. Currently, there is no available crystal structure of OAMB, hence, homology modeling using GPCR-I-TASSER [47] was employed to produce a rational 3D structure of the protein. GPCR-I-TASSER automatically selects the putative templates by threading through their GPCR PDB library followed by template-based fragment assembly to construct a full-length model.

After identifying the different essential oil components of the mabolo fruit, ligand 3D structures were prepared using the MarvinSketch software [48] prior to docking simulations. Octopamine, the putative ligand, was selected as the positive control in the molecular docking studies. Using the binding affinity and the binding site of OA as reference, the potential of other ligands, i.e., isolated compounds from the mabolo fruit, as biopesticide candidates were assessed.

### 3.3. Molecular Dynamics: Protein Conformational Ensemble

The bilayer simulation of OAMB was prepared using the default parameters of the Charmm-GUI [49] web server. The lipid bilayer was composed of 1:1 DPPC (1,2-Dipalmitoyl-sn-glycero-3-phosphocholine) and DPPE (1,2-Dipalmitoyl-sn-glycero-3-phospho-ethanolamine) in the upper and lower leaflets. Additionally, 80 K${}^{+}$ and 99 Cl${}^{-}$ were automatically added to emulate a 0.15 M KCl ionic environment. In preparation of the molecular dynamics simulation, the protein and the lipid bilayer system were parameterized using the AMBER ff14SB [50] and AMBER lipid14 [51] forcefields, respectively. K${}^{+}$ and Cl${}^{-}$ ions were parametrized using the monovalent ion parameters for explicit solvents [52].

Molecular dynamics simulation was performed on the protein-bilayer system using the Nanoscale Molecular Dynamics (NAMD 2.10) software (Theoretical and Computational Biophysics group at the Beckman Institute, University of Illinois at Urbana-Champaign, Urbana, IL, USA) [53,54,55]. After energy minimization was performed, the system was heated from 0 K to 300 K, followed by a 2 ns equilibration prior to the production protocol. The simulation parameters for the production were set at a temperature of 300 K, at constant *NVT* using Langevin dynamics [56]. Additionally, the particle mesh Ewald method [57] was used to evaluate the long-range electrostatic interactions while the bonds with hydrogen atoms were constrained using the SHAKE algorithm [58]. After a 100 ns production run, an ensemble consisting of 100 different protein conformations were generated from the entire simulation using the clustering analysis of the cpptraj [59] module of the AMBERTools 15 package [60].

### 3.4. Ensemble Docking

After obtaining 100 representative protein conformations from the molecular dynamics simulation, docking was performed via AutoDock Vina [61] using octopamine and the isolated compounds as ligands. Important protein-ligand interactions are usually located in the transmembrane and extracellular regions of GPCRs [62,63,64]. As such, the search space was set around the centroid of the transmembrane helices with grid dimensions 40 Å × 40 Å × 40 Å, which also includes the extracellular loops. Binding affinities of the ligands were obtained from the scoring function of Vina.

The inhibition constant ($K_{i}$) can be calculated from the free energy relation $K_{i}=exp\left(\frac{\Delta G}{RT}\right)$. Since the ensemble docking consists of 100 different protein conformations per ligand, we report the average binding energy and inhibition constants for each ligand at 95% confidence level ($\bar{x}\pm \frac{1.96\sigma }{\sqrt{n}}$), where $\bar{x}$ is the mean, σ is the standard deviation, *n* is the population equal to 100, and the 1.96 multiplier at 95% confidence level.

## 4. Conclusions

Mabolo, an indigenous tree of the Philippines, bears an edible reddish-orange fruit with a characteristic smell owing it to the presence of several volatile metabolites. An interesting attribute of the mabolo fruit is the abundance of essential oils that are used for various applications. This study taps on the potential of mabolo to be a source of effective plant-based biopesticides targeting OAMB. In this study, we employed untargeted metabolomics with ensemble docking to screen mabolo fruit metabolites against *Drosophila melanogaster* octopamine receptor in mushroom bodies (OAMB). Extraction of mabolo essential oil components mostly yielded esters with methyl benzoate being the most abundant. Results from the docking studies show significant interactions between the ligands and the residues of the binding region, i.e., Val69, Val99, Met103, Cys104, Ser107, Trp532, Gly559, Trp560, and Asn562. OAMB seems to favor complexing with ligands containing an aryl group because of the numerous aromatic residues in the vicinity of the binding region. As such, interactions with aromatic amino acids could be explored in formulating functionally modified molecules with increased binding affinity than that of the metabolites found in mabolo. Overall, these small aromatic compounds from mabolo are promising motifs for biopesticides that target OAMB. Live insect assays and in vitro studies to verify our in silico results are ongoing.

## Figures and Tables

**Table 1:** Isolated essential oil components by gas chromatography with their retention time (in min) and their percent abundance in the extracted oils.

Compounds Identified	Retention Time (min)	Relative Abundance
Methyl benzoate	8.2	53.4
Benzyl butanoate	11.5	15.2
Benzyl alcohol	11.6	9.8
Ethyl butanoate	3.4	8.5
Butyl butanoate	5.1	6.0
Methyl butanoate	3.0	5.6
Butyl benzoate	11.4	1.4

**Table 2:** Average binding energies of the OAMB-ligand complexes formed and their calculated inhibition constants computed using the Gibbs free energy relation $K_{i}=e^{\frac{\Delta G}{RT}}$. The mean binding affinities of the ligands, at 95% confidence level, to OAMB were obtained from the ensemble docking procedure. Statistical data presented is computed at 95% confidence interval. $\bar{x}\pm \frac{1.96\sigma }{\sqrt{n}}$ where *n* = 100.

Ligand	Average Binding Affinity (kcal·mol${}^{-1}$)	Average $K_{i}$ (μmolar)
Octopamine	−5.18 ± 0.07	191.9 ± 27.4
Benzyl butanoate	−6.03 ± 0.09	54.0 ± 12.3
Butyl benzoate	−6.02 ± 0.01	47.6 ± 7.2
Methyl benzoate	−5.61 ± 0.07	94.2 ± 15.0
Benzyl alcohol	−4.93 ± 0.06	280.3 ± 37.9
Butyl butanoate	−4.88 ± 0.06	304.1 ± 32.4
Ethyl butanoate	−4.41 ± 0.06	657.8 ± 65.2
Methyl butanoate	−4.06 ± 0.05	1140.7 ± 93.6

## Abbreviations

The following abbreviations are used in this manuscript:

cAMP	cyclic Adenosine monophosphate
GABA	γ-aminobutyric acid
GC-MS	Gas chromatography - Mass spectroscopy
GPCR	G-Protein Coupled Receptors
MD	Molecular dynamics
OA	Octopamine
OAMB	Octopamine Receptor in Mushroom Bodies
OAR	Octopamine Receptors
PDB	Protein Databank
